# Long-term follow-up of papillary and follicular thyroid carcinomas with bone metastasis

**DOI:** 10.1371/journal.pone.0173354

**Published:** 2017-03-09

**Authors:** Jen-Der Lin, Shu-Fu Lin, Szu-Tah Chen, Chuen Hsueh, Chia-Lin Li, Tzu-Chieh Chao

**Affiliations:** 1 Division of Endocrinology and Metabolism, Departments of Internal Medicine, Chang Gung Memorial Hospital, Chang Gung University, Taoyuan County, Taiwan, R.O.C.; 2 Department of Pathology, Chang Gung Memorial Hospital, Chang Gung University, Taoyuan County, Taiwan, R.O.C.; 3 Healthy Aging Research Center, Chang Gung University, Taoyuan County, Taiwan, R.O.C.; 4 Department of General Surgery, Chang Gung Memorial Hospital, Chang Gung University, Taoyuan County, Taiwan, R.O.C.; Seoul National University College of Pharmacy, REPUBLIC OF KOREA

## Abstract

The aims of this study were to investigate papillary and follicular thyroid carcinomas with bone metastasis in various clinical presentations and to determine the prognostic factors after multimodality treatment. A retrospective analysis was performed of 3,120 patients with papillary and follicular thyroid carcinoma. Of these patients, 131 (including 97 women, 71.8%) were diagnosed with bone metastasis and underwent follow-up at the Chang Gung Medical Center. Patients with bone metastasis were categorized into two groups. Group A was comprised of patients who were diagnosed with bone metastasis either before thyroidectomy or within 6 months of the initial thyroidectomy (90 patients, 68.7%). Group B was comprised of patients with bone metastasis who received a diagnosis 6 months post-thyroidectomy in the follow-up period (41 patients, 31.3%). After a mean follow-up period of 8.4 ± 7.0 years, there were 88 deaths (67.2%) attributed to thyroid cancer and 13 patients (9.9%) achieved disease-free status. A multivariate analysis showed that older age, early diagnosis, and brain metastasis were each associated with a poor prognosis. The difference in disease-specific mortality rates between groups A and B was significant (p < 0.0001). In conclusion, papillary and follicular thyroid cancers with bone metastasis have a high rate of mortality. Despite this high mortality, 9.9% patients still had an excellent response to treatment.

## Introduction

Thyroid cancer is the most common malignancy of the endocrine system, with an increasing incidence in the past three decades. Well-differentiated thyroid cancers, including papillary and follicular carcinomas, comprise approximately 85% to 90% of all thyroid cancers and usually have an excellent prognosis following appropriate treatment [1,2]. However, 3%-20% of patients develop distant metastasis during the treatment or follow-up [3–6]. Distant metastases from thyroid carcinomas typically appear in the lungs and bone [5]. Compared to lung involvement, patients with bone metastasis generally have a worse prognosis [5,7]. The incidence of bone metastasis is 1%-7% in papillary thyroid carcinoma and 7%-20% in follicular thyroid carcinoma [8]. The mechanisms underlying the tendency of well-differentiated thyroid carcinoma to leading to bone metastasis is not entirely clear [6,9]. Bone metastases of well-differentiated thyroid carcinoma are most often osteolytic lesions. Skeletal metastases can cause pain, pathologic fractures, and spinal cord compression. However, there are also clinically silent bone metastases. The treatments for bone metastasis of well-differentiated thyroid carcinoma include radioactive iodide (^131^I), surgical resection, external beam radiation therapy, arterial embolization, systemic bisphosphonates or chemotherapy, and percutaneous image-guided treatments [8,10,11]. With these therapeutic modalities, the prognosis in patients with bone metastatic disease remains poor, with a 10-year survival rate <50%. However, long-term survival has been demonstrated in a small proportion of patients [5,7]. The use of prognostic factors in risk stratification and development of personalized therapy is necessary for this population. The purpose of this study was to investigate the prognostic factors and long-term outcomes in patients diagnosed with papillary and follicular thyroid carcinomas with bone metastasis.

## Subjects and methods

### Study design

**Fig 1:**
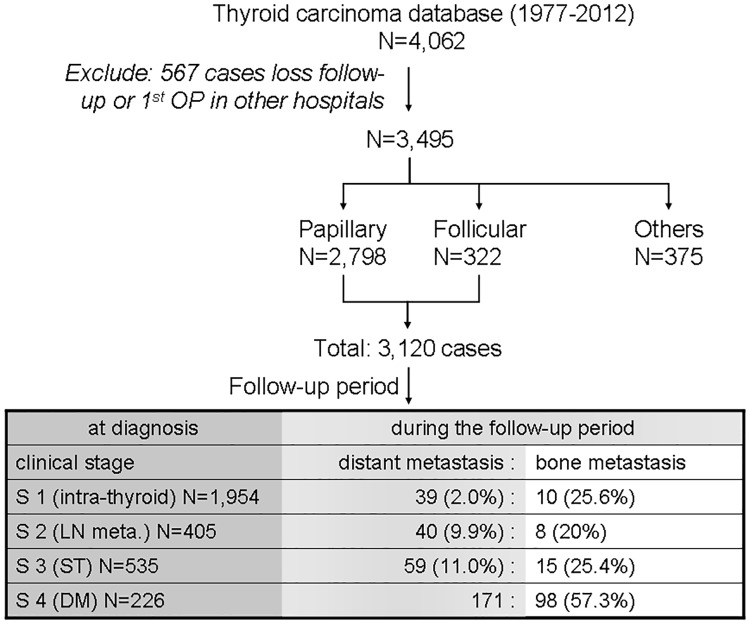
Cases of papillary and follicular thyroid carcinoma with bone metastasis enrolled from the thyroid cancer patient database of the Chang Gung Memorial Hospital. The clinical staging used was as follows: stage I, tumor confined to the thyroid; stage II, lymph node (LN) metastasis; stage III, soft tissue (ST) involvement; and stage IV, distant metastasis (DM).

We reviewed 4,062 cases of thyroid cancer in our database from between 1977 and 2012. A total of 567 cases were excluded either because patients did not receive follow up care at our hospital (479 cases) or because the first thyroidectomy took place at other hospitals and no detailed information was available (88 cases) (Fig 1). Among 3,120 patients with papillary and follicular thyroid carcinoma, 131 (94 women, 71.8%) were diagnosed with bone metastasis and underwent follow-up care at the Chang Gung Medical Center in Linkou, Taiwan. At the time of initial thyroidectomy, 33 cases were clinically diagnosed as clinical stage I, II, or III (Fig 1). An additional 98 cases were diagnosed as distant metastasis, including bone metastasis, at the time of thyroidectomy. Inclusion criteria for patients in this study were histopathologically proven papillary or follicular thyroid carcinomas with bone metastasis proven using tissue biopsy or imaging with ^131^I, computed tomography (CT), or magnetic resonance imaging (MRI). Patients with insufficient data were excluded from the study.

### Patient grouping

Of the 131 patients with bone metastasis, 87 (66.4%) underwent a total thyroidectomy. For the other 44 patients, only a partial thyroidectomy (including subtotal, lobectomy, or biopsy) was performed. Reasons for partial thyroidectomy included patient refusal of complete thyroidectomy, advanced locoregional invasion, or poor health of the patient rendering them unsuitable for the procedure. Patients were categorized into two groups. Group A included patients who were diagnosed with bone metastasis either before thyroidectomy or within 6 months of the initial thyroidectomy (90 patients, 68.7%). Of the patients in group A, 29 patients (22.1%) had bone metastasis confirmed by tissue biopsy before thyroidectomy. Of the remaining 61 patients in group A, 45 presented with ^131^I-avid lesions in distant metastases. There were 6 cases of distant metastases and elevated thyroglobulin (Tg), as diagnosed using CT imaging. Seven cases were diagnosed with bone or lung metastases with elevated Tg via chest or skeletal radiography. An additional 3 cases were diagnosed via bone scintigraphy, including 1 case, which was diagnosed using MRI at the same time. Group B included patients with bone metastasis who received a diagnosis 6 months post-thyroidectomy in the follow-up period (41 patients, 31.3%). Among the 41 patients in group B, 33 were diagnosed with ^131^I-avid lesions in the distant metastases. Five cases of distant metastases were diagnosed using CT, 2 cases were diagnosed using bone scintigraphy, and 1 case was diagnosed using MRI.

The tumors in all of the patients in the study were staged using the American Joint Committee on Cancer- tumor node metastasis (AJCC-TNM) criteria (7^th^ edition) [12]. All thyroid carcinomas were pathologically classified according to the World Health Organization (WHO) criteria [13]. A second primary malignancy was diagnosed in patients with thyroid cancer based on the malignancy diagnosis codes 140–208.91 in the International Classification of Diseases, 9th Revision (ICD-9) clinical modification format. Patients with malignancy required diagnostic validation by at least two specialists, evaluated based on patient medical examination records, laboratory and imaging results, and histological or cytological analyses. Patients diagnosed with malignancies were categorized into different groups according to the anatomical organ system involved.

### Additional diagnosis and therapy

Approximately 4–6 weeks after thyroidectomy, thyroid remnant ablation was recommended for patients with high-risk papillary and follicular thyroid carcinoma. The ^131^I ablation dose for most patients was 1.1–3.7 GBq (30–100 mCi). Low-risk patients, defined as those with no residual cancer tissue detected during the operation, or no distant metastasis suspected via other imaging techniques (radiography or neck ultrasonography), received 30 mCi of ^131^I. In contrast, 100 mCi of ^131^I was administered to patients with high-risk or intermediate-risk, including those with lymph node metastasis or soft tissue invasion. If preoperative distant metastasis was diagnosed, such as in group A of this study, or if residual cancer was detected, 5.6–7.4 GBq (100–200 mCi) of ^131^I was administered. A whole body scan (WBS) was performed 1 week after ^131^I administration using a dual-head gamma camera (Dual Genesys, ADAC, USA) equipped with a high-energy collimator, as detail described previously [5,14]. Subsequently, L-T_4_ treatment was initiated to decrease thyroid stimulating hormone (TSH) levels without inducing clinical thyrotoxicosis. The therapeutic target level of TSH was 0.01–0.35 uIU/mL. Cases in which the foci of ^131^I uptake extended beyond the thyroid bed were classified as persistent disease or metastasis. Patients with bone metastasis received increased therapeutic doses of ^131^I at 5.6–7.4 GBq (100–200 mCi). Hospital isolation was arranged for patients requiring doses exceeding 1.1 GBq. A WBS was performed 2 weeks after the administration of the higher therapeutic dose of ^131^I. Depending on the clinical indications, the noninvasive examinations performed included the following radiological and nuclear investigations: CT, MRI, bone scintigraphy, thallium-201 (^201^Tl) scintigraphy, and fluoro-18-deoxyglucose positron emission tomography-CT (PET-CT). PET-CT has been used for evaluation of thyroid cancer in our hospital since 2007.

At the end of 2014, patient mortality was categorized into thyroid cancer-related mortality and total mortality. Survival of patients with thyroid cancer was categorized according to the modified American Thyroid Association (ATA) guidelines into an excellent response group and a non-remission group [15]. The excellent response group was defined as having a negative imaging finding including an ^131^I scan, suppressed Tg levels <0.12 ng/mL, or stimulated Tg levels <1 ng/mL.

### Statistical analysis

All data are expressed as the mean ± standard error. Univariate and multivariate statistical analyses were performed to determine the significance of various factors using the Kaplan-Meier method and log-rank test [16]. *P*-values < 0.05 were considered statistically significant. In addition, survival rates were calculated using the Kaplan-Meier method and were compared using the Breslow and Mantel-Cox tests. This study was approved by the Chang Gung Medical Foundation Institutional Review Board (102-3433B). Because of the retrospective nature of this study, the requirement for informed consent was waived. The statistical software SPSS 21.0 and GraphPad Prism 5.0 were used in this study.

## Results

Table 1 shows the clinical features of patients with papillary and follicular thyroid carcinoma with bone metastasis categorized into two groups. Factors such as the age, sex, tumor size, therapeutic modalities (including surgical method), accumulated ^131^I dose, and external radiotherapy were not statistically different between the two groups. Group B patients showed a higher incidence of papillary thyroid carcinoma, a less-advanced TNM stage, a lower median postoperative Tg value, and longer follow-up periods than those in group A. Among the patients receiving ^131^I therapy, 98 patients (74.8%) had ^131^I -avid lesions in the distant metastatic sites. The percentage of ^131^I -avid patients was not significantly different between groups A and B (*p* = 0.312). Among 87 patients who underwent total thyroidectomy, with or without lymph node dissection, 78 (89.7%) had ^131^I -avid metastatic lesions. In contrast, 28 of 44 (63.6%) patients who underwent partial thyroidectomy presented with ^131^I-avid lesions.

**Table 1:** Clinical features of papillary or follicular thyroid cancer with bone metastasis in different group.

Clinical Characteristic	All patients	Group A vs. B		*P* value
		Group A	Group B	
Patient number (%)	131 (100.0)	90 (68.7)	41 (31.3)	
Gender, Female (%)	94 (71.8)	65 (72.2)	29 (70.7)	0.861
Age at diagnosis (year)	57.4 ± 15.0	59.1 ± 13.2	53.7 ± 18.0	0.058
(range)	(11–87)	(11–87)	(11–87)	
Mean tumor size (cm)	4.1 ± 2.9	3.8 ± 2.6	4.6 ± 3.3	0.151
(range)	(0.5–18.0)	(0.5–11.1)	(0.7–18.0)	
**Median post-operative serum Tg level after 1 month (ng/mL)**	**300**	**520**	**29**	**0.008**
**(range)**	**(0.0–141970.0)**	**(1.7–141970.0)**	**(0.0–7890.0)**	
Multifocality	15 (11.5)	10 (11.1)	5 (12.2)	0.857
Operative method				
Total thyroidectomy	87 (66.4)	56 (62.2)	31 (75.6)	0.132
Less than total thyroidectomy	44 (33.6)	34 (37.8)	10 (24.4)	
**Histology**				
**Papillary**	**79 (60.3)**	**45 (50.0)**	**34 (82.9)**	**<0.001**
Follicular	52 (39.7)	45 (50.0)	7 (17.1)	
**TNM stage**				
**Stage I**	**10 (7.6)**	-	**10 (24.4)**	**<0.001**
Stage II	19 (14.5)	12 (13.3)	7 (17.1)	
Stage III	8 (6.1)	-	8 (19.5)	
Stage IV	94 (71.8)	78 (86.7)	16 (39.0)	
**Follow-up period (year)**	**8.4 ± 7.0**	**6.6 ± 6.4**	**12.2 ± 6.8**	**<0.001**
Post-operative ^131^I accumulative dose (mCi)	451.4 ± 480.8	412.2 ± 470.3	537.4 ± 492.2	0.200
(range)	(0.0–2787.1)	(0.0–2000.0)	(0.0–2787.1)	
Radiation therapy	68 (51.9)	51 (56.7)	17 (41.5)	0.106
^131^I avid lesion	98 (74.8)	65 (72.2)	33 (80.5)	0.312
Brain metastasis	8 (6.1)	7 (7.8)	1 (2.4)	0.184
Total mortality	96 (73.3)	73 (81.1)	23 (56.1)	0.237
**Cancer mortality**	**88 (67.2)**	**69 (76.7)**	**19 (46.3)**	**0.003**
**Excellent response**	**13 (9.9)**	**5 (5.6)**	**8 (19.5)**	**0.001**

Number (%)

Among the 131 cases included in the study, 52 (39.7%) were diagnosed as follicular thyroid carcinoma. Of the 52 follicular thyroid carcinomas, there was only 1 minimally invasive variant and 1 case with poorly differentiated components. Of the 79 cases of papillary thyroid carcinoma, there were 24 follicular variants, 5 with poorly differentiated components, and 3 tall cell variants. Patients with follicular thyroid carcinoma had a notably higher incidence of bone metastasis compared with those who had papillary thyroid carcinoma (59/322 patients, 16% vs. 79/2,798 patients, 2%, respectively). A comparison between follicular and papillary thyroid carcinomas with bone metastasis showed a higher incidence of follicular carcinoma in group A and an older average patient age in the follicular group. Patients in the follicular thyroid carcinoma group also had a more advanced TNM stage, and higher disease-specific and total mortality rates. Among the patients in group A, 29 were diagnosed with bone metastasis before thyroidectomy, including 14 patients who presented with vertebral metastasis, 4 patients with skull metastasis, and the remaining patients with metastatic involvement of the sternum, the ischium, and the extremities. Of the 131 patients, 19 required additional neck dissection for locoregional recurrence, including 7 patients who underwent two operations. A total of 68 (51.9%) patients underwent external radiotherapy (Table 1).

**Fig 2:**
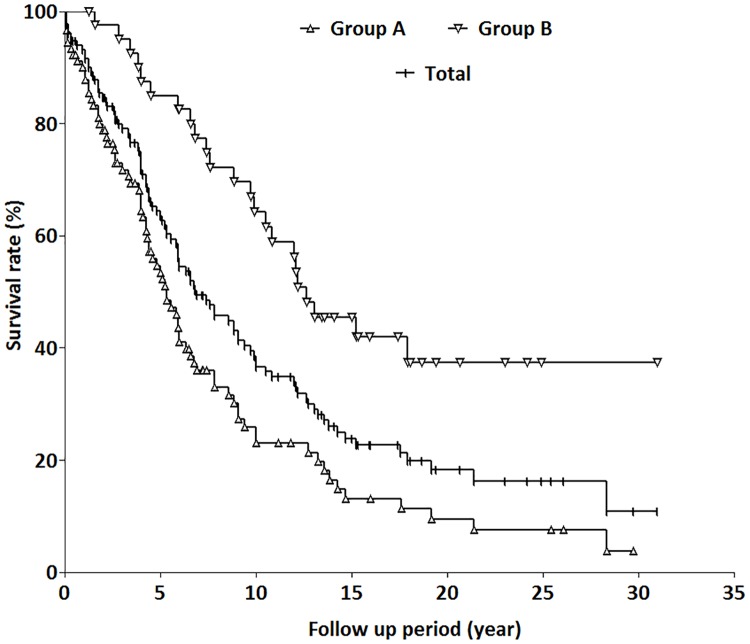
Thyroid cancer-specific survival rates for the three groups of patients with bone metastasis. The disease-specific survival rates for groups A, B, and for all patients included in the study were 53.4%, 85.0%, and 63.5% at 5 years; 22.9%, 64.2%, and 36.7% at 10 years; 9.4%, 37.3%, and 18.2% at 20 years; and 3.7%, 37.3%, and 10.8% at 30 years, respectively. The difference in disease-specific mortality rates between groups A and B was significant difference (*p* < 0.0001).

The peak disease incidence in the male group occurred 10 years earlier than in the female group (41–50 vs. 51–60 years, respectively). After a mean follow-up of 8.4 ± 7.0 years, 88 patients (67.2%) died of thyroid cancer, and 13 patients (9.9%) achieved an excellent response status. Fig 2 shows the thyroid cancer-specific survival rates for patients by group and combined. The disease-specific survival rates for groups A, B, and for all patients in the study were 53.4%, 85.0%, and 63.5% at 5 years; 22.9%, 64.2%, and 36.7% at 10 years; 9.4%, 37.3%, and 18.2% at 20 years; and 3.7%, 37.3%, and 10.8% at 30 years, respectively. The difference in disease-specific mortality rates between groups A and B was significant (*p* < 0.0001). The disease- specific survival rate in group B was significantly higher than that in group A (Fig 2).

**Fig 3:**
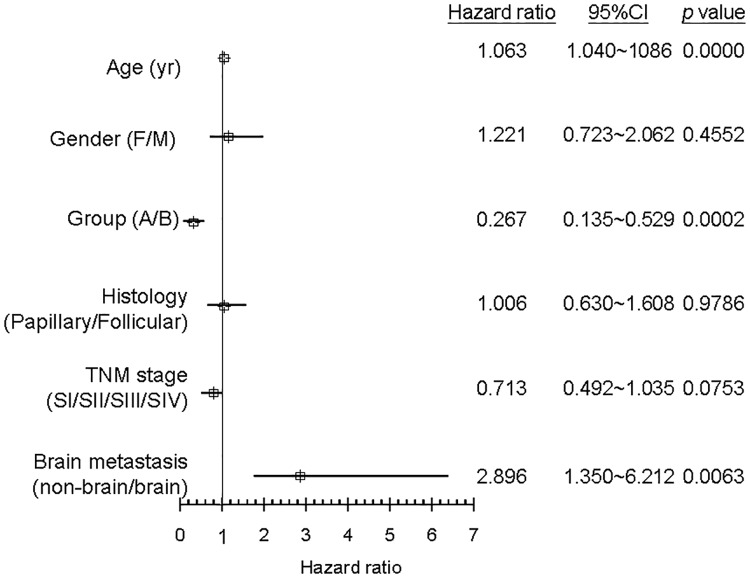
Multivariate analysis using cox proportional hazards regression model for survival and mortality.

Table 2 shows the univariate analysis of thyroid cancer mortality. Disease-specific mortality was higher among patients with an early diagnosis, women, older patients, the follicular thyroid carcinoma group, patients with advanced TNM stage, and those with brain metastasis or invasion. Multivariate analysis showed that age, early diagnosis, and brain metastasis were statistically significant factors and Hazard ratio for cancer mortality (Fig 3).

**Table 2:** Clinical features of papillary or follicular thyroid cancer with bone metastasis in thyroid cancer mortality or non-cancer mortality.

Clinical Characteristic	All Patients	Cancer Mortality vs. Non-cancer Mortality		*P* value
		Cancer Mortality	Non-cancer Mortality	
Patient number	131 (100.0)	88 (67.2)	43 (32.8)	
**Group A**	**90 (68.7)**	**69 (76.7%)**	**21 (23.3%)**	**0.001**
**Group B**	**41 (31.3)**	**19 (46.3%)**	**22 (53.7%)**	
**Gender, Female**	**94 (71.8)**	**68 (77.3)**	**26 (60.5)**	**0.045**
**Age at diagnosis (year)**	**57.4 ± 15.0**	**60.5 ± 13.1**	**51.0 ± 16.7**	**0.001**
**Mean tumor size (cm)**	**4.1 ± 2.**	**4.7 ± 3.3**	**3.1 ± 1.7**	**0.011**
Median post-operative serum Tg level after 1 month (ng/mL)	300	472	81.8	0.300
(range)	(0.0–141970.0)	(1.4–141970.0)	(0.0–61845.0)	
Multifocality	15 (11.5)	9 (10.2)	6 (14.0)	0.529
Operative method				
Total thyroidectomy	87 (66.4)	56 (63.6)	31 (72.1)	0.336
Less than total thyroidectomy	44 (33.6)	32 (36.4)	12 (27.9)	
**Histology**				
**Papillary**	**79 (60.3)**	**43 (54.4)**	**36 (45.6)**	**<0.001**
**Follicular**	**52 (39.7)**	**45 (86.5)**	**7 (13.5)**	
**TNM stage**				
**Stage I**	**10 (7.6)**	**2 (2.3)**	**8 (18.6)**	**0.002**
**Stage II**	**19 (14.5)**	**10 (11.4)**	**9 (20.9)**	
**Stage III**	**8 (6.1)**	**5 (5.7)**	**3 (7.0)**	
**Stage IV**	**94 (71.8)**	**71 (80.7)**	**23 (53.5)**	
**Follow-up period (year)**	**8.4 ± 7.0**	**6.6 ± 5.3**	**11.9 ± 8.6**	**<0.001**
Post-operative ^131^I accumulative dose (mCi)	451.4 ± 480.8	433.8 ± 449.5	491.1 ± 543.2	0.612
**Radiation therapy**	**68 (51.9)**	**54 (61.4)**	**14 (32.6)**	**0.002**
^131^I avid lesion	98 (74.8)	63 (71.6)	35 (81.4)	0.225
2^nd^ primary cancer	10 (7.6)	5 (5.7)	5 (11.6)	0.229
**Brain metastasis**	**8 (6.1)**	**8 (9.1)**	-	**0.041**

Number (%)

## Discussion

Distant metastasis of well-differentiated thyroid cancer has been reported in 1.4%-9% of patients at the time of initial diagnosis, depending on the case selection [3,5,17]. Among the cases of distant metastasis, bone metastasis occurred less frequently than lung metastasis. However, bone metastasis was associated with a poorer prognosis as compared to lung metastasis [18,19]. In our study, bone metastases in papillary and follicular thyroid carcinomas presented at different times and were diagnosed either via imaging techniques or histological tissue analysis. The diagnosis and treatment of well-differentiated thyroid cancers have changed in recent decades. As in a previous study [20], distant metastasis concurrent with the diagnosis of well-differentiated thyroid cancer was unusual and had a poor prognosis. Our results are similar to those of the study from the Memorial Sloan-Kettering Cancer Center, in which the 5-year overall survival rate and disease-specific survival rate were 65% and 68%, respectively [20]. Extrapulmonary metastasis, including bone metastasis, and follicular thyroid carcinoma indicated a worse prognosis than papillary thyroid carcinoma.

Compared with the other groups, group A patients had a higher rate of cancer mortality (76.7%). Most patients who were diagnosed with bone metastasis before thyroidectomy presented with skeletal pain or neurological disorders, as described in a previous study [17]. Over half of our patients underwent external radiotherapy for symptom relief and to reduce the tumor size. Although patients in this group were treated for a neurological deficiency with a poor prognosis, 5 patients (5.6%) responded well to the surgical removal of the bone metastatic tissue followed by ^131^I treatment. In recent studies, preoperative neurological deficit was significantly associated with worse overall survival and preoperative embolization was significantly associated with improved overall survival [21,22]. The disease-specific survival of patients with papillary and follicular thyroid carcinoma is fairly good, although inadequate treatment of bone metastasis can result in severe morbidities. Therefore, radical surgery for metastatic bone lesions should be considered, especially for those with otherwise favorable prognostic factors of cancer mortality [7].

In our study, there were more patients with bone metastasis in the papillary thyroid carcinoma group than in the follicular thyroid carcinoma group. As shown in previous studies, hematogenous spreading and distant metastasis were more common in follicular thyroid carcinoma [23,24]. Additionally, in our study, patients with follicular thyroid carcinoma were older and had larger tumors, similar to previous observations [25]. Compared with patients with papillary thyroid carcinoma, a higher percentage of patients with follicular thyroid carcinoma were diagnosed with bone metastasis before or during thyroidectomy, and they showed a higher rate of disease-specific mortality. Preoperative fine needle aspiration cytology of classic papillary thyroid carcinoma usually shows characteristic features of intranuclear grooving and inclusion bodies in the nucleus [24,26]. In contrast, follicular thyroid carcinoma must be diagnosed as having capsular or vascular invasion in the final histopathological assessment. This is the main reason for the delayed diagnosis of follicular thyroid carcinoma.

Among our patients, there were 8 cases with intracranial involvement, including 4 that were diagnosed histopathologically. Compared with other malignancies, there were few well-differentiated thyroid carcinomas with intracranial involvement [27,28]. As shown previously, intracranial involvement, including brain metastasis and intracranial invasion from the scalp, were indicators of a poor prognosis relative to those with bone metastasis without brain involvement [29]. Surgical resection and multidisciplinary adjuvant treatment were critical for improving outcomes in patients with intracranial metastasis from thyroid cancer. All of our patients with intracranial involvement died of thyroid cancer within a median follow-up period of 5 years.

In the long-term follow-up period of this study, we found that patients diagnosed with bone metastasis during the follow-up had a better prognosis than those diagnosed at the initial thyroidectomy. Patients with follicular thyroid carcinoma had a worse prognosis than those with papillary thyroid carcinoma. Achievement of an excellent response after treatment was significantly higher in group B, possibly due to less advanced disease at the time of thyroidectomy, TNM staging, and a lower percentage of patients with follicular thyroid carcinoma. The limitations of this study include changing and inconsistent therapeutic and diagnostic modalities, owing to the long-term follow-up period. In addition, most well-differentiated thyroid carcinoma with bone metastasis did not have tissue proven disease. Basing the diagnosis on clinical features and ^131^I-avid lesions, there was a low possibility of including cancers of non-thyroid origin. In conclusion, papillary and follicular thyroid carcinomas with bone metastasis have a high rate of mortality. However, we found that the 5-year survival rates exceeded 5 years in different clinical presentation groups. Aggressive surgical treatment to remove metastatic lesions and postoperative ^131^I therapy are recommended in such cases.

## Supporting information

S1 Minimal Data This is minimal data supporting information. (XLS)

## References

[1] Grogan RH, Kaplan SP, Cao H, Weiss RE, Degroot LJ, Simon CA, et al A study of recurrence and death from papillary thyroid cancer with 27 years of median follow-up. Surgery. 2013; 154: 1436–1446. 10.1016/j.surg.2013.07.008 24075674

[2] Untch BR, Palmer FL, Ganly I, Patel SG, Michael Tuttle R, Shah JP, et al Oncologic outcomes after completion thyroidectomy for patients with well-differentiated thyroid carcinoma. Ann Surg Oncol. 2014; 21: 1374–1378. 10.1245/s10434-013-3428-1 24366419

[3] Sampson E, Brierley JD, Le LW, Rotstein L, Tsang RW. Clinical management and outcome of papillary and follicular (differentiated) thyroid cancer presenting with distant metastasis at diagnosis. Cancer. 2007; 110: 1451–1456. 10.1002/cncr.22956 17705176

[4] Cho SW, Choi HS, Yeom GJ, Lim JA, Moon JH, Park DJ, et al Long-term prognosis of differentiated thyroid cancer with lung metastasis in Korea and its prognostic factors. Thyroid. 2014; 24: 277–286. 10.1089/thy.2012.0654 23758653 PMC3926138

[5] Lin JD, Hsueh C, Chao TC. Long-term follow-up of the therapeutic outcomes for papillary thyroid carcinoma with distant metastasis. Medicine. 2015; 94(26): e1063 10.1097/MD.0000000000001063 26131826 PMC4504566

[6] Phay JE, Ringel MD. Metastatic mechanisms in follicular cell-derived thyroid cancer. Endocr Relat Cancer. 2013; 20: R307–319. 10.1530/ERC-13-0187 24036131 PMC4196318

[7] Nakayama R, Horiuchi K, Susa M, Watanabe I, Watanabe K, Tsuji T, et al Clinical outcome after bone metastasis (BM) surgery in patients with differentiated thyroid carcinoma (DTC): a retrospective study of 40 cases. Jpn J Clin Oncol. 2014; 44: 918–925. 10.1093/jjco/hyu099 25104791

[8] Wexler JA. Approach to the thyroid cancer patient with bone metastases. J Clin Endocrinol Metab. 2011; 96: 2296–2307. 10.1210/jc.2010-1996 21816796

[9] Croucher PI, McDonald MM, Martin TJ. Bone metastasis: the importance of the neighbourhood. Nat Rev Cancer. 2016; 16: 373–386. 10.1038/nrc.2016.44 27220481

[10] Satcher RL, Lin P, Harun N, Feng L, Moon BS, Lewis VO. Surgical management of appendicular skeletal metastases in thyroid carcinoma. Int J Surg Oncol. 2012; 2012: 417086 10.1155/2012/417086 23304478 PMC3530792

[11] Cazzato RL, Bonichon F, Buy X, Godbert Y, de Figuereido BH, Pointillart V, et al Over ten years of single-institution experience in percutaneous image-guided treatment of bone metastases from differentiated thyroid cancer. Eur J Surg Oncol. 2015; 41: 1247–1255. 10.1016/j.ejso.2015.06.005 26136221

[12] Edge SE, Byrd DR, Carducci MA, Compton CA. Eds 2009 AJCC Cancer Staging Manual. Seventh edition Springer-Verlag, New York, New York.

[13] Delellis RA, Lloyd RV, Heitx PU, Eng C. Pathology and genetics of tumors of endocrine organs In World Health Organization of Tumours. IARC, Lyon, 2004; pp: 73–76.

[14] Liu FH, Kuo SF, Hsueh C, Chao TC, Lin JD. Postoperative recurrence of papillary thyroid carcinoma with lymph node metastasis. J Surg Oncol. 2015; 112: 149–154. 10.1002/jso.23967 26175314 PMC5034820

[15] Haugen BR, Alexander EK, Bible KC, Doherty GM, Mandel SJ, Nikiforov YE, et al 2015 American Thyroid Association Management Guidelines for Adult Patients with Thyroid Nodules and Differentiated Thyroid Cancer: The American Thyroid Association Guidelines Task Force on Thyroid Nodules and Differentiated Thyroid Cancer. Thyroid. 2016; 26: 1–133. 10.1089/thy.2015.0020 26462967 PMC4739132

[16] Zhang DD, Zhou XH, Freeman DH, Freeman JL. A non-parametric method for the comparison of partial areas under ROC curves and its application to large health care data sets. Stat Med. 2002; 21: 701–715. 11870811 10.1002/sim.1011

[17] Farooki A, Leung V, Tala H, Tuttle RM. Skeletal-related events due to bone metastases from differentiated thyroid cancer. J Clin Endocrinol Metab. 2012; 97: 2433–2439. 10.1210/jc.2012-1169 22564664

[18] Muresan MM, Olivier P, Leclère J, Sirveaux F, Brunaud L, Klein M, et al Bone metastases from differentiated thyroid carcinoma. Endocr Relat Cancer. 2008; 15: 37–49. 10.1677/ERC-07-0229 18310274

[19] Ito Y, Kudo T, Kobayashi K, Miya A, Ichihara K, Miyauchi A. Prognostic factors for recurrence of papillary thyroid carcinoma in the lymph nodes, lung, and bone: analysis of 5,768 patients with average 10-year follow-up. World J Surg. 2012; 36: 1274–1278. 10.1007/s00268-012-1423-5 22270990

[20] Nixon IJ, Whitcher MM, Palmer FL, Tuttle RM, Shaha AR, Shah JP, et al The impact of distant metastases at presentation on prognosis in patients with differentiated carcinoma of the thyroid gland. Thyroid. 2012; 22: 884–889. 10.1089/thy.2011.0535 22827579 PMC3714454

[21] Sellin JN, Suki D, Harsh V, Elder BD, Fahim DK, McCutcheon IE, et al Factors affecting survival in 43 consecutive patients after surgery for spinal metastases from thyroid carcinoma. J Neurosurg Spine. 2015; 23: 419–428. 10.3171/2015.1.SPINE14431 26140400

[22] Ramadan S, Ugas MA, Berwick RJ, Notay M, Cho H, Jerjes W, et al Spinal metastasis in thyroid cancer. Head Neck Oncol. 2012; 4: 39 10.1186/1758-3284-4-39 22730910 PMC3466148

[23] Sugino K, Kameyama K, Nagahama M, Kitagawa W, Shibuya H, Ohkuwa K, et al Follicular thyroid carcinoma with distant metastasis: outcome and prognostic factor. Endocr J. 2014; 61: 273–279. 24420337 10.1507/endocrj.ej13-0437

[24] Lin JD, Chao TC. Follicular thyroid carcinoma: diagnosis to treatment. Endocrine J. 2006; 53: 441–448. 16807500 10.1507/endocrj.kr-77

[25] Lang BH, Wong KP, Cheung CY, Wan KY, Lo CY. Evaluating the prognostic factors associated with cancer-specific survival of differentiated thyroid carcinoma presenting with distant metastasis. Ann Surg Oncol. 2013; 20: 1329–1335. 10.1245/s10434-012-2711-x 23104708 PMC3599207

[26] Albores-Saavedra J, Wu J. The many faces and mimics of papillary thyroid carcinoma. Endocr Pathol. 2006; 17: 1–18. 16760576 10.1385/ep:17:1:1

[27] Chiu AC, Delpassand ES, Sherman SI. Prognosis and treatment of brain metastases in thyroid carcinoma. J Clin Endocrinol Metab. 1997; 82: 3637–3642 10.1210/jcem.82.11.4386 9360519

[28] Lee HS, Yoo H, Lee SH, Gwak HS, Shin SH. Clinical characteristics and follow-up of intracranial metastases from thyroid cancer. Acta Neurochir (Wien). 2015; 157: 2185–2194. 26476828 10.1007/s00701-015-2611-5

[29] Henriques de Figueiredo B, Godbert Y, Soubeyran I, Carrat X, Lagarde P, Cazeau AL, et al Brain metastases from thyroid carcinoma: a retrospective study of 21 patients. Thyroid. 2014; 24: 270–276. 10.1089/thy.2013.0061 23734630

